# BDNF and Cortisol in the Diagnosis of Cocaine-Induced Depression

**DOI:** 10.3389/fpsyt.2022.836771

**Published:** 2022-03-15

**Authors:** Francina Fonseca, Joan Ignasi Mestre-Pinto, Rocío Rodríguez-Minguela, Esther Papaseit, Clara Pérez-Mañá, Klaus Langohr, Margherita Barbuti, Magí Farré, Marta Torrens, NEURODEP GROUP

**Affiliations:** ^1^Addiction Research Group (GRAd), Neuroscience Research Program, Hospital del Mar Medical Research Institute (IMIM), Barcelona, Spain; ^2^Institut de Neuropsiquiatria i Addiccions, Hospital del Mar, Barcelona, Spain; ^3^Department of Medicine and Life Sciences (MELIS), Universitat Pompeu Fabra, Barcelona, Spain; ^4^Clinical Pharmacology Department, Hospital Universitari Germans Trias i Pujol (IGTP), Badalona, Spain; ^5^Department of Pharmacology, Therapeutics and Toxicology, Universitat Autònoma de Barcelona (UAB), Cerdanyola del Vallès, Spain; ^6^Department of Statistics and Operations Research, Universitat Politècnica de Catalunya - BarcelonaTech, Barcelona, Spain; ^7^Integrative Pharmacology and Systems Neuroscience Research Group, Neuroscience Research Programme, Hospital del Mar Medical Research Institute (IMIM), Barcelona, Spain; ^8^Psychiatry 2 Unit, Department of Clinical and Experimental Medicine, University Hospital of Pisa, Pisa, Italy; ^9^Department of Psychiatry and Legal Medicine, Universitat Autònoma de Barcelona (UAB), Cerdanyola del Vallès, Spain

**Keywords:** depression, cocaine use disorder, cortisol, dual diagnosis, brain derived neurotrophic factor (BDNF)

## Abstract

**Background:**

Major depressive disorder (MDD) and cocaine use disorder (CUD) are related with disability and high mortality rates. The assessment and treatment of psychiatric comorbidity is challenging due to its high prevalence and its clinical severity, mostly due to suicide rates and the presence of medical comorbidities. The aim of this study is to investigate differences in brain derived neurotrophic factor (BDNF) and cortisol plasmatic levels in patients diagnosed with CUD-primary-MDD and CUD-induced-MDD and also to compare them to a sample of MDD patients (without cocaine use), a sample of CUD (without MDD), and a group of healthy controls (HC) after a stress challenge.

**Methods:**

A total of 46 subjects were included: MDD (*n* = 6), CUD (*n* = 15), CUD-primary-MDD (*n* = 16), CUD-induced-MDD (*n* = 9), and 21 HC. Psychiatric comorbidity was assessed with the Spanish version of the Psychiatric Research Interview for Substance and Mental Disorders IV (PRISM-IV), and depression severity was measured with the Hamilton Depression Rating Scale (HDRS). Patients were administered the Trier Social Stress Test (TSST) before and after the biological measures, including BDNF, and cortisol levels were obtained.

**Results:**

After the TSST, Cohen's *d* values between CUD-primary-MDD and CUD-induced-MDD increased in each assessment from 0.19 post-TSST to 2.04 post-90-TSST. Pairwise differences among CUD-induced-MDD and both MDD and HC groups had also a large effect size value in post-30-TSST and post-90-TSST. In the case of the BDNF concentrations, CUD-primary-MDD and CUD-induced-MDD in post-90-TSST (12,627.27 ± 5488.09 vs.17,144.84 ± 6581.06, respectively) had a large effect size (0.77).

**Conclusion:**

Results suggest a different pathogenesis for CUD-induced-MDD with higher levels of cortisol and BDNF compared with CUD-primary-MDD. Such variations should imply different approaches in treatment.

## Introduction

Mental and substance use disorders (SUD) are related with 7% of the global burden of disease as measured in disability-adjusted life years (DALYs) and increasing significant mortality rates (1). In women, DALYs are generally associated with major depression disorder (MDD) while, in men, addictive disorders are more prevalent (1).

The assessment and treatment of psychiatric comorbidity is challenging because of its high prevalence (2, 3) and clinical severity, mostly due to suicide rates and the presence of medical comorbidities (4–6). Moreover, it is essential to distinguish between primary and induced MDD as they vary with respect to prognosis, relapse risk (7), and response to antidepressants (8). Traditionally, the implications of induced depression have been minimized. Some clinicians believe that it involved a mild syndrome that could revert with substance abstinence. As a consequence, induced MDD was not treated unless symptoms persisted (9). Subsequent research, however, demonstrates that relapse risk is even greater in the case of induced MDD than primary (7). Moreover, some longitudinal studies demonstrate that patients with an initial diagnosis of induced MDD after some years developed a primary one (10). With respect to antidepressants, a number of studies and reviews indicate differences in response depending on the type of depression with worse response to serotonin selective reuptake inhibitors (SSRI) for induced MDD (11, 12).

At the international level, cocaine is one of the most widely used illicit drugs. The 2021 United Nations Office on Drugs and Crime World Report estimated that around 20 million individuals aged 15–64 years (0.4%) had consumed cocaine during 2019 (13). Its use is associated with medical and psychopathological comorbidities, for example, increased risk of blood-borne infections (such as HIV and hepatitis C); elevated rates of mortality; and increased prevalence of mental health disorders, mainly depression, psychotic episodes, and suicide attempts (14). There are no approved pharmacological treatments for cocaine use disorder (CUD), and only some weak effects from psychotherapy are described (14, 15).

Individuals taking cocaine are reported to present a high risk of depression (16). The euphoria induced by acute cocaine use can induce a cycle of self-treatment of depressive symptoms, leading to a severe presentation of both CUD and MDD.

Due to the reasons mentioned above, clinicians are faced with having to distinguish between CUD-primary-MDD and CUD-induced-MDD in cocaine consumers. In a similar manner to psychiatric disorders, there are, however, no valid biomarkers for their correct identification. The diagnosis of MDD (induced/primary) is based on the subjective identification of clinical symptoms, and there are no clear standards for differential diagnosis. A recent study comparing DSM-5 criteria only found that “changes in weight or appetite” had a differing prevalence among the two disorders (17). The identification of biological markers in depressive disorders and SUD could help in the process of accurate diagnosis. MDD and CUD share some neurobiological pathways (18), for example, brain derived neurotrophic factor (BDNF) and cortisol levels. They are described as being able to assist in the diagnosis and identification of outcome predictors in MDD not associated with substance use (19).

The hypothalamic-pituitary-adrenal (HPA) axis is involved in the pathogenesis of MDD (20). Traditional studies observed a blunted stress response in MDD following a stress challenge, such as the Trier Social Stress Test (TSST) (21). Other studies, however, found a hyper-response but only in patients with severe depression (22). The HPA axis could also play a role in both CUD and MDD. In a study performed in cocaine-dependent patients, an infusion of intravenous cocaine was associated with adrenocorticotropic hormone (ACTH) and cortisol levels and depressive symptoms measured with the Hamilton Depression Rating Scale (HDRS) (23).

The BDNF belongs to the peptide family involved in neural plasticity, neurogenesis, and neural survival (24). It also has a key role in acute and chronic responses to substances of abuse. For instance, in a prospective study, BDNF plasma concentrations were associated in cocaine addiction with relapse risk in early recovery (25). Another study demonstrated that plasma concentrations of BDNF during early cocaine abstinence correlated with withdrawal syndrome and craving (26). Moreover, BDNF is associated with a number of psychiatric syndromes, including depression (27). The neurotrophic hypothesis of depression postulates that low levels of BDNF could induce atrophy at limbic structures and prefrontal cortex (28), whereas antidepressant treatment increases BDNF levels in depressed patients (29). With respect to dual diagnosis patients, BDNF levels are shown to present differences in samples from cocaine addicts with and without depression. Those with a comorbid diagnosis of cocaine addiction and depression, irrespective of being primary or induced, show lower BDNF levels (30).

Both MDD and SUD are complex diseases that result from changes in differing physiological systems. Thus, to better understand their pathophysiologies, the combined study of different systems and networks is required. In this regard, a recent paper by Chen et al. finds that combining the results of serum BDNF, cortisol, and interferon-gamma could help in making an accurate diagnosis of MDD (31).

At present, it is crucial to perform accurate diagnoses of CUD-primary-MDD and CUD-induced-MDD. As the monoamine hypothesis of depression is proven insufficient to explain differences between both types of MDD (32), it is essential to investigate the involvement of different systems in dual depression. We, therefore, carried out this study aimed at investigating differences in BDNF and cortisol plasmatic levels in patients diagnosed with CUD-primary-MDD and CUD-induced-MDD and also to compare them with a sample of MDD patients (without cocaine use), a sample of CUD (without MDD), and a group of healthy controls (HC) before and after a stress challenge.

## Methods

### Subjects and Study Design

In this cohort study, the sample included subjects with (i) MDD (*n* = 6), (ii) CUD (*n* = 15), (iii) CUD and primary MDD (*n* = 16), and (iv) CUD and induced MDD (*n* = 9). All patients were recruited at the addiction treatment facilities of the Parc Salut Mar Institute of Neuropsychiatry and Addiction in Barcelona, Spain.

Inclusion criteria were aged >18 years, Caucasian origin, body mass index 19–29 Kg/m^2^, and the absence of any other psychiatric disorder and/or SUD other than MDD and/or CUD. In patients with primary/induced MDD the most recent episode had to be in remission, and the 17-item HDRS (33, 34) score <6. In the CUD groups, subjects had to have maintained at least 4 weeks of substance abstinence prior to the trial as confirmed by random urine controls. Cognitive or language limitations that precluded assessments, pregnancy or breast-feeding, use of anti-inflammatory drugs or monoamine oxidase inhibitors, and any medical problem that might interfere in the study procedures were considered exclusion criteria.

HCs (*n* = 21) were included from a database of subjects willing to participate in medical research projects at the Pharmacology Unit of the Hospital del Mar Institute of Medical Research (IMIM), Barcelona, Spain. In the HC group, the exclusion criteria were any Axis I psychiatric disorder, family history of depression, and any SUD except nicotine.

After basal clinical and psychiatric assessment, both patients and HCs participated in the stress experimental sessions with the TSST.

Subjects were admitted to the IMIM Clinical Research Unit facilities at 08.00. Those presenting nicotine addiction were treated during the experimental session with patches according to their nicotine daily dose. A urine sample was collected for drug testing (Instant-View®, Multipanel 10 Test Drug Screen, Alfa Scientific Designs Inc., Poway, CA, USA). Participants were required to be drug-free before the experimental session. The subjects remained sitting/lying in a calm laboratory environment during the session with restricted social interactions. The TSST was performed at 13.00 hours. This was carried out to (i) assure a similar waking time for all participants the day of the test, (ii) control activities that could affect HPA axis functioning, (iii) avoid heterogeneity of the cortisol response, and (iv) assure a period of rest before the protocol was administered (35, 36).

### Clinical and Psychiatric Assessments

At baseline assessment, a closed-ended questionnaire was used to record participants' sociodemographic characteristics, family history, medical assessment, history of substance use, and previous psychiatric treatment. Psychiatric diagnoses were performed according to DSM-IV-TR criteria with the Spanish version of the Psychiatric Research Interview for Substance and Mental Disorders IV (PRISM-IV) (37). PRISM was specifically designed to deal with the issues of psychiatric diagnosis in SUD patients. It helps differentiate primary disorders, SUD, and the expected effects of intoxication and withdrawal. Diagnoses obtained through the PRISM interview are demonstrated to have good-to-excellent validity and test–retest reliability for primary-MDD and substance-induced MDD (38). In the MDD patients, depression severity was evaluated with the Spanish version of the HDRS (34).

### TSST

The TSST is an acute stress test that consists of two tasks: public speaking and a mathematical task (39). Participants were asked to deliver a speech about their holidays or favorite book/film to a group of experts in nonverbal communication. After 5 min, three individuals (the audience) unfamiliar to the participant entered the room. The participant was instructed by one audience member (the spokesperson) to begin his/her prepared speech (without notes) for 5 min. If the individual paused, he/she was instructed by the spokesperson to continue. At the end of the speaking task, the individual was instructed to serially subtract 17 from 3,164 or 2,043 (randomly) as quickly and accurately as possible. The mental mathematic recitation continued for 5 min, at the end of which the spokesperson instructed the individual to stop, and the audience left the procedure room. Both tests were videorecorded. The experimental assessment was conducted before the test (pre-TSST); immediately after (post-TSST); and after 30 (post30-TSST), 60 (post60-TSST), and 90 min (post90-TSST). At the same time points, physiological and biochemical data were obtained. The TSST is proven useful in inducing acute stress response even in patients with CUD (40).

### Biological Measurements

Heart rate (HR), systolic blood pressure (SBP), diastolic blood pressure (DBP), respiratory rate (RR), and temperature were monitored by Dash 3,000 monitor (GE, Wisconsin, USA) at different times: before the test (pre-TSST); immediately after (post-TSST); and after 30 (post30-TSST), 60 (post60-TSST), and 90 min (post90-TSST). At the same time points, blood samples were collected from the subjects.

#### Cortisol

To assess cortisol levels, 5 ml of peripheral blood sample was centrifuged at 4,000 rpm for 10 min. The serum obtained was frozen at −20°C until analysis was conducted by electrochemiluminescence, using an Immulite-2000 XPi analyzer (Siemens).

#### BDNF

BDNF was obtained before (pre-TSST), immediately after (post-TSST), and at 90 min (post90-TSST). Five milliliters of peripheral blood sample was centrifuged at 4,000 rpm for 10 min. The serum obtained was frozen at −20°C until analysis, which was performed with 500 microliters of serum by ELISA and the kit Human BDNF Quantikine ELISA Kit of R&D-Vitro SA and polyclonal antibodies.

### Statistical Analysis

A descriptive analysis of all variables of interest was carried out separately in each of the study groups. For this purpose, the mean, median, standard deviation, and range were calculated. Repeated-measure ANOVA models were used to analyze the intragroup and intergroup changes of both the cortisol and BDNF concentrations. The models included group condition as a main factor in addition to all two- and three-way interactions. The computation of simultaneous confidence intervals and adjusted *p*-values to guarantee a family-wise error rate of 0.05 was based on the multivariate *t* distribution of the vector of test statistics.

Next, one-way ANOVA models were fitted to compare the study groups with the mean of the variables. The model assumptions (homoscedasticity and normally distributed residuals) were checked with residual plots and the Levene (homoscedasticity) and Kolmogorov-Smirnov tests, respectively. If assumptions held and group differences were statistically significant, the Bonferroni test was applied for the *post hoc* pairwise comparisons. Cohen's *d* was used to quantify the effect size of the pairwise differences among study groups (small: *d* ≤ 0.20; medium: *d* ≥ 0.50; large: *d* ≥ 0.80; very large: *d* ≥ 1.30). Cohen's *d* is a standardized score, analogous to a *z* score. Following Cohen's effect size conventions, only differences higher than a medium effect size (*d* ≥ 0.50) were considered of relevance.

All data were analyzed using the IBM Corp. Released 2013 IBM SPSS Statistics for Windows, Version 22.0 (Armonk, NY: IBM Corp.). In the case of the group comparisons, statistical significance was set at 0.05 (to protect against type I errors), and for model assumption tests at 0.1 (to protect against type II errors).

### Ethics Statement

The clinical protocol was approved by the local Research Ethical Committee CEIC-Parc de Salut Mar, Barcelona, Spain (2009/3494/I and 2012/4751/I), and the study was conducted in accordance with the Declaration of Helsinki and Spanish laws concerning clinical research. Volunteers were financially compensated. All subjects gave written informed consent prior to their participation in the study.

## Results

### Demographic and Clinical Characteristics

A total of 67 subjects were included in the study to assess possible differences in BDNF and cortisol levels during the TSST. The main sociodemographic and clinical characteristics of the sample are described in Table 1. The final groups were 21 HC, 6 MDD, 9 CUD-induced-MDD, 16 CUD-primary-MDD, and 15 CUD.

**Table 1:** Sociodemographic and clinical characteristics of the sample at baseline (*n* = 67).

	**HC (*N* = 21)**	**MDD (*N* = 6)**	**CUD-induced-MDD** **(*N* = 9)**	**CUD-primary-MDD** **(*N* = 16)**	**CUD** **(*N* = 15)**
Sex (Male) N (%)	14 (66.7)	5 (83.3)	7 (77.8)	13 (81.3)	12 (80)
Age (Mean ± SD)	32.6 ± 4.8	45.7 ± 13.2	37.7 ± 11.4	44.8 ± 7.8	38.0 ± 9.5
Civil status (% Single)	10 (47.6)	2 (33.3)	6 (66.7)	6 (37.5)	10 (66.7)
Work status (% Employed)	10 (47.6)	2 (33.3)	3 (33.3)	7 (43.8)	4 (26.7)
Depression (MDD)					
HDRS (Mean ± SD)	0.57 ± 1.21	1.17 ± 1.83	0.56 ± 0.73	1.38 ± 1.09	0.73 ± 1.33
Age of onset first induced-MDD (Mean ± SD)	-	-	33.3 ± 11.8		-
Age of onset first primary-MDD (Mean ± SD)	-	36.2 ± 10.9	-	37.5 ± 6.4	-
Number of episodes (Mean ± SD)	-	1.8 ± 1.0	3.2 ± 2.1	2.5 ± 1.9	-
Family history of depression (%)	-	5 (83.3)	4 (44.4)	11 (68.8)	5 (33.3)
Current antidepressant treatment (%)	-	5 (83.3)	4 (44.4)	10 (62.5)	2 (13.3)
Age of cocaine problematic use	-	-	26.3 ± 9.0	29.3 ± 6.8	26.3 ± 7.1
Nicotine use disorder (%)	-	-	7 (77.8)	12 (75)	11 (73.3)

*HC, healthy controls; MDD, major depression disorder; CUD, cocaine use disorder; SD, standard deviation*.

More than 76% of the total sample were single men aged >32 years. All groups had low HDRS scores, and the depressed groups had more than one MDD episode with a similar age of onset. Family history of depression and current treatment with antidepressants were also more prevalent in these groups. In the CUD groups, the age of onset of problematic cocaine use was very similar, and current nicotine use was >73%.

Five out of six subjects in the MDD group were on varying types of antidepressants. In the CUD-induced and primary-MDD groups, the majority of patients were also on antidepressant treatment. The CUD-primary-MDD patients were treated with SSRIs although in the induced-MDD group other types of antidepressants were prescribed. Types of antidepressants in the study groups are described in (Supplementary Table S1).

### Trier Social Stress Test (TSST)

#### Biological Measures

Changes in HR, SBP, DBP, and RR before and after the TSST are depicted in Supplementary Table S2. CUD and HC presented significant changes over time in HR and DBP without differences in the rest of the groups.

#### Cortisol

**Figure 1:**
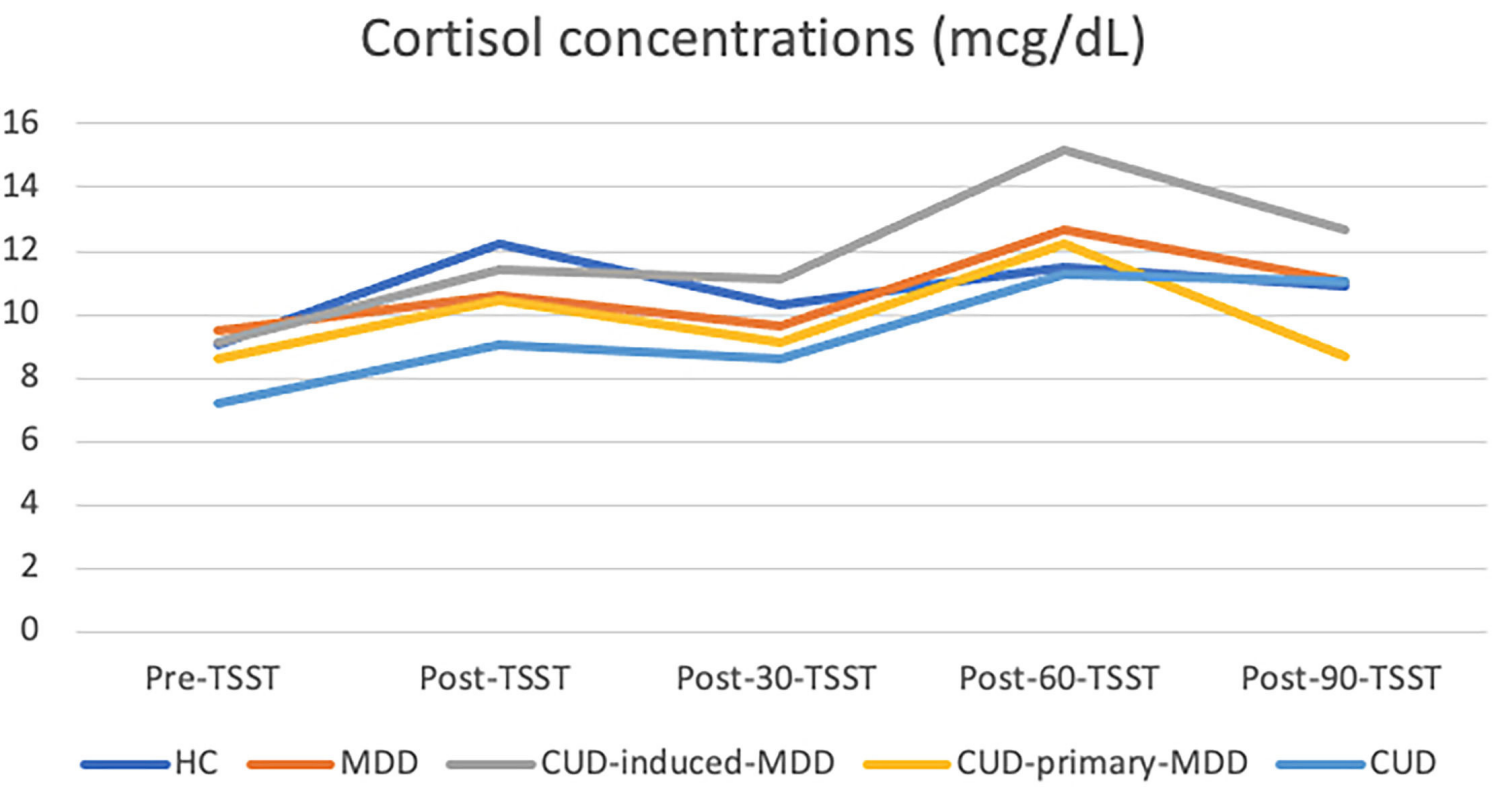
Mean cortisol concentrations during Trier social stress test (TSST). HC, healthy control (*n* = 21); MDD, major depression disorder (*n* = 6); CUD, cocaine use disorder (*n* = 15), CUD-primary-MDD, (*n* = 16), CUD-induced-MDD (*n* = 9).

All groups showed a similar response pattern during the TSST follow-ups with the CUD-induced-MDD presenting the highest cortisol concentrations in post-60 TSST and the CUD-primary-MDD the lowest cortisol concentrations in post-90 TSST (Figure 1).

One-way ANOVA tests yielded *p*-values >0.117 when comparing groups in each assessment (Table 2). A paired *T*-test showed significant within-group changes in cortisol concentrations over time except in the MDD group with the lowest range of change [9.48 ± 2.7–12.7 ± 3.47].

**Table 2:** Mean cortisol concentrations during Trier social stress test (TSST).

**Cortisol mcg/dL**	**Pre-TSST Mean ±SD**	**Post-TSST Mean ±SD**	**Post-30-TSST Mean ±SD**	**Post-60-TSST Mean ±SD**	**Post-90-TSST Mean ±SD**
HC (*N* = 21)	9.06 ± 3.67	12.24 ± 4.52^*^	10.27 ± 3.95^*^	11.45 ± 3.36	10.87 ± 3.72^*^
MDD (*N* = 6)	9.48 ± 2.7	10.62 ± 1.35	9.63 ± 3.55	12.7 ± 3.47	11.05 ± 2.55
CUD-induced-MDD (*N* = 9)	9.09 ± 5	11.4 ± 6.95^*^	11.1 ± 5.15^*^	15.18 ± 2.17^*^	12.64 ± 1.55
CUD-primary-MDD (*N* = 16)	8.63 ± 2.17	10.48 ± 3.09	9.11 ± 2.6^*^	12.25 ± 4.3	8.69 ± 2.11^*^
CUD (*N* = 15)	7.16 ± 3.03	9.03 ± 3.06^*^	8.6 ± 2.99	11.27 ± 4.25^*^	11.01 ± 6.12
One-way ANOVA	*p* = 0.453	*p* = 0.253	*p* = 0.461	*p* = 0.117	p = 0.160

* *Paired T-Test < 0.05*.

*TSST, Trier social stress test; HC, healthy controls; MDD, major depression disorder; CUD, cocaine use disorder; SD, standard deviation*.

After the TSST, Cohen's *d* values between CUD-primary-MDD and CUD-induced-MDD increased in each assessment from 0.19 post-TSST to 2.04 post-90-TSST. Pairwise differences among CUD-induced-MDD and both MDD and HC groups had also a large effect size value post-30-TSST and post-90-TSST (Table 3).

**Table 3:** Effect size coefficient (Cohen's d) pairwise comparisons of cortisol concentrations in each assessment.

		**MDD**	**CUD-induced -MDD**	**CUD-primary -MDD**	**CUD** **(*N* = 15)**
Pre-TSST	HC (*N* = 21)	0.12	0.01	0.14	0.56
	MDD (*N* = 6)		0.09	0.37	0.79
	CUD-induced-MDD (*N* = 9)			0.13	0.50
	CUD-primary-MDD (*N* = 16)				0.56
Post-TSST	HC	0.40	0.16	0.44	0.81
	MDD		0.14	0.05	0.59
	CUD induced MDD			0.19	0.49
	CUD primary MDD				0.47
Post-30-	HC	0.17	0.19	0.34	0.47
TSST	MDD		0.32	0.18	0.33
	CUD induced MDD			0.54	0.64
	CUD primary MDD				0.18
Post-60-	HC	0.37	1.22	0.21	0.05
TSST	MDD		0.90	0.11	0.35
	CUD induced MDD			0.79	1.08
	CUD primary MDD				0.23
Post-90-	HC	0.05	0.54	0.70	0.03
TSST	MDD		0.80	1.06	0.01
	CUD induced MDD			2.04	0.33
	CUD primary MDD				0.51

*Cohen's effect size: small (d > 0.20), medium (d > 0.50), large (d > 0.80), and very large (d > 1.30). HC, healthy controls; MDD, major depression disorder; CUD, cocaine use disorder; TSST, Trier social stress test*.

#### BDNF

**Figure 2:**
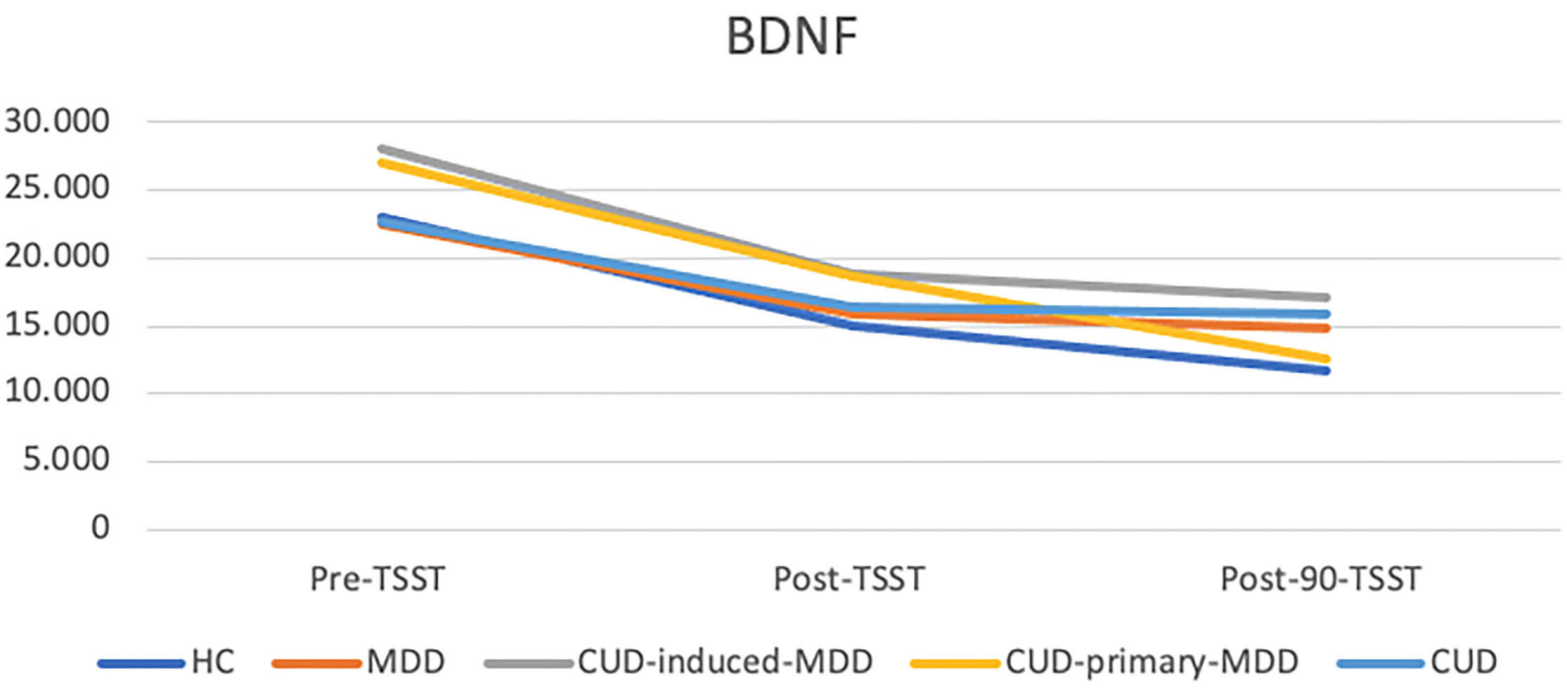
Mean BDNF concentrations during Trier social stress test (TSST). HC, healthy control (*n* = 21); MDD, major depression disorder (*n* = 6); CUD, cocaine use disorder (*n* = 15), CUD-primary-MDD, (*n* = 16), CUD-induced-MDD (*n* = 9).

All groups showed a similar decreasing pattern in BDNF concentrations during TSST follow-ups. The CUD-induced-MDD group had the highest BDNF concentrations in each assessment (Figure 2).

One-way ANOVA demonstrated statistical differences post-90-TSST (*p* = 0.032); no differences, however, were reported when performing Bonferroni's *post-hoc* comparisons (Table 4). A paired *T*-test showed significant within-group changes in BDNF concentrations over time except in the MDD group.

**Table 4:** Mean BDNF concentrations during Trier social stress test (TSST).

**BDNF**	**Pre-TSST**	**Post-TSST**	**Post-90-TSST**
HC (*N* = 21)	23,053.15 ± 4,818.28	14,993.06 ± 3,882.56^*^	11,782.55 ± 4,138.4^*^
MDD (*N* = 6)	22,510.41 ± 5619.91	15,912.88 ± 3,456.21	14,872.87 ± 2,471.91
CUD-induced-MDD (*N* = 9)	28,040.91 ± 5,287.74	18,854.29 ± 4,662.07^*^	17,144.84 ± 6,581.06^*^
CUD-primary-MDD (*N* = 16)	27,076.84 ± 8,457.33	18,735.27 ± 6,045^*^	12,627.27 ± 5,488.09^*^
CUD (*N* = 15)	22,739.07 ± 5,673.17	16,423.37 ± 4,950.92^*^	15,888.35 ± 5,050.1^*^
One-way ANOVA	*p* = 0.082	*p* = 0.125	*p* = 0.032

* *Paired T-Test < 0.05*.

*HC, healthy controls; MDD, major depression disorder; CUD, cocaine use disorder; TSST, Trier social stress test*.

Table 5 depicts the pairwise differences among the CUD-induced-MDD and HC groups that had the largest effect size value in the three assessments with values >0.94. The difference in BDNF concentrations between CUD-primary-MDD and CUD-induced-MDD post-90-TSST (12,627.27 ± 5488.09 vs.17,144.84 ± 6581.06, respectively) also had a large effect size (0.77).

**Table 5:** Effect size coefficient (Cohen's d) pairwise comparisons of BDNF concentrations in each assessment.

	**BDNF**	**MDD**	**CUD-induced -MDD**	**CUD-primary -MDD**	**CUD** **(*N* = 15)**
Pre-TSST	HC (*N* = 21)	0.11	1.01	0.61	0.06
	MDD (*N* = 6)		1.02	0.58	0.04
	CUD-induced-MDD (*N* = 9)			0.13	0.96
	CUD-primary-MDD (*N* = 16)				0.60
Post-TSST	HC	0.24	0.94	0.76	0.33
	MDD		0.69	0.51	0.11
	CUD-induced-MDD			0.02	0.50
	CUD-primary-MDD				0.42
Post-90-	HC	0.80	1.08	0.18	0.91
TSST	MDD		0.42	0.46	0.22
	CUD-induced-MDD			0.77	0.22
	CUD-primary-MDD				0.62

*Cohen's effect size: small (d > 0.20), medium (d > 0.50), large (d > 0.80), and very large (d > 1.30). HC, healthy controls; MDD, major depression disorder; CUD, cocaine use disorder; TSST, Trier social stress test*.

## Discussion

The most important finding of this study is the different response observed after a stress challenge (TSST) in the levels of cortisol and BDNF in primary and induced depression. As the diagnosis of depression is based on clinical criteria, sometimes with suboptimal rates of validity and accuracy, the detection of measurable biomarkers has implications in the study of the pathophysiology of depression and the introduction of effective treatments.

As previously reported, the monoamine theory of depression cannot completely explain the pathogenesis of induced depressions (32), and other physiological systems should be studied. Stress is related to both MDD (41, 42) and SUD (43, 44), and in turn, cortisol is associated with stress. In our study, patients diagnosed with CUD-induced-MDD showed higher levels of cortisol after an acute stress challenge compared with CUD-primary-MDD ones. Such differences could indicate that varying mechanisms are involved in these two types of depression.

Moreover, when analyzing BDNF plasma levels, we observed similar differences with higher concentrations of BDNF at 90 min after the TSST in the CUD-induced-MDD compared with the CUD-primary-MDD and MDD without cocaine use. Traditional research describes lower levels of BDNF in depressive patients (28). In our sample, higher levels of BDNF at 90 min were observed in the CUD and the CUD-induced-MDD groups; surprisingly, the HC group showed the lowest BDNF concentrations. Varying concentrations and level changes depending on the type of depression could explain differences in therapeutical response to treatment between induced and primary depressive disorders and the lack of response of some of them.

The use of cortisol and BDNF levels as markers to differentiate cocaine-induced and primary depressions could help in the design of personalized treatments. They would permit the correct selection of antidepressant, thus avoiding prolonged periods before patient response to treatment (45). Nevertheless, reviews and meta-analysis have not clearly defined whether there are differences in BDNF level increases depending on the antidepressant evaluated (29). With respect to BDNF levels in CUD, one study evaluated changes in plasma concentrations during detoxification. It was observed that chronic cocaine use was associated with lower levels of BDNF, and during detoxification, the levels increased, correlating with cocaine craving (26). In our sample, at baseline, subjects with higher concentrations of BDNF were those with CUD and MDD, either primary or induced, although findings were nonsignificant. In addition, after the stress challenge, BDNF levels decreased in all groups although maintaining the higher levels those of the CUD-induced-MDD group.

Our findings do not signify a causal model, and it was not possible to clarify whether the differences in cortisol and BDNF levels were primary or secondary to induced/primary depression. Could a previously disrupted HPA axis be a marker of depression risk? Another study reported that patients with CUD presented a previous childhood history (parent neglect), higher scores in depression severity (measured by the SCL-90), and greater levels of ACTH and cortisol in plasma than HCs. The authors concluded that early life events (neglect and poor attachment to parents) influenced HPA axis function, and additionally, such individuals presented increased vulnerability to depression and substance use (46). In this regard, another study evaluated salivary cortisol and hemodynamic data (BP and HR) response to the TSST in subjects prenatally exposed to cocaine. When comparing these subjects to nonexposed ones, it was observed that the former presented higher rates of cortisol levels before and after the TSST, a finding that suggests an impaired response to stress in subjects prenatally exposed to cocaine (47). Another explanation could be the presence of untreated depression as a risk factor for CUD. In a study evaluating an animal model, depressed rats administered more cocaine than nondepressed ones showed higher concentrations of BDNF at the prelimbic cortex (48). In this regard, it should be noted that our participants were either abstinent or in remission from the last depressive episode. Previous research has not described differences depending on the time patients were abstinent from psychostimulant drugs and TSST response (40). For instance, the fact that patients did not present depressive symptoms (HDRS <6) hindered results being presented as a state marker although they could be interpreted as a trait maker. In other words, CUD-induced and CUD-primary-MDD should have different stress responses, and levels of BDNF and cortisol correlate with impaired stress responsiveness in these types of patients. The TSST has been useful in other kinds of research to discriminate different stress responses, for example, in young people exposed to prenatal cocaine (47).

More comprehensive knowledge regarding dual depression biomarkers and the differences between primary and induced depression are essential to introduce effective treatment, particularly as the improvement of depressive symptoms requires at least 4 weeks after commencement of antidepressant therapy. Moreover, previous research, in accordance with the present results, demonstrates differential neurobiological processes underlying induced and primary MDD (32, 49) suggesting poor outcomes with SSRI antidepressants (8, 12). For instance, in the case of depression with lower levels of BDNF, the first line treatment should be those antidepressants that are shown to raise BDNF levels, such as agomelatine (50). Martinotti reports that, in patients with MDD, a correlation between depressive symptom improvement and BDNF serum concentrations was observed after 2 weeks of agomelatine treatment. A recent review also (29) focused on the effects of antidepressants in BDNF levels and found that, in general, antidepressants increased the levels of BDNF. It was not possible, however, to identify the differential effects by type of antidepressant; a better description of depression phenotypes is probably called for.

There are several limitations to this study. First, the sample size was small for all groups. Indeed, the strict inclusion criteria made it difficult to find pure cocaine/depressed-only patients. For this reason, although our results suggest biochemical differences between CUD-primary-MDD and CUD-induced-MDD, such findings should be confirmed by the analysis of a larger set of samples. A second limitation is that the MDD patients were under remission, and differences could, therefore, be underestimated. The decision to include patients in remission was made due to ethical reasons in order not to expose individuals with depression to a stress situation (even in a controlled environment). This means that the differences observed should be considered as trait markers or risk factors to develop depression and not clinical depression itself. In previous research, the TSST is useful to discriminate risk factors in stress response for healthy controls (51), the general population (52), and patients with active depression (53). In addition, most of the depressed group participants were under antidepressant treatment, which could influence BDNF levels as previously described (29). Finally, the small sample size also hindered a proper evaluation of the effect of gender on the results. Previous authors observe differences in the cortisol response to acute stress between genders after an acute stress challenge (54, 55). Studies evaluating the results between men and women are therefore essential to adapt interventions.

Reliable biomarkers are needed to detect and diagnose depression subtypes. One strength of the study is that these molecules could be analyzed routinely. Future studies investigating their involvement in the outcome and response to treatment are warranted.

## Data Availability Statement

The raw data supporting the conclusions of this article will be made available by the authors, without undue reservation.

## Ethics Statement

The studies involving human participants were reviewed and the clinical protocol was approved by the local Research Ethical Committee CEIC-Parc de Salut Mar, Barcelona, Spain (2009/3494/I and 2012/4751/I) and the study was conducted in accordance with the Declaration of Helsinki and Spanish laws concerning clinical research. Volunteers were financially compensated. All subjects gave written informed consent prior to their participation in the study. The patients/participants provided their written informed consent to participate in this study.

## Author Contributions

MT was the principal investigator of the grants supporting the research. MT and MF were responsible for the study concept and design. MT, MF, JM-P, and FF designed the protocol. FF, RR-M, CP-M, and EP selected the participants. JM-P, CP-M, EP, and MF conducted the TSS test sessions. JM-P and KL performed the statistical analysis and FF, JM-P, MF, and MT interpreted findings. FF, JM-P, and MB wrote the initial draft of the manuscript. MT and MF provided critical revision of the manuscript for key intellectual content. All authors have read and agreed to the published version of the manuscript.

## Funding

This work was supported by grants from the Instituto de Salud Carlos III–ISCIII Red de Trastornos Adictivos 2016 (RD16/0017/0010 and RD16/0017/0003); Fondo de Investigación Sanitaria (FIS) (PI09/02121, PI12/01838, PI16/00603); National R + D+I and funded by the Instituto de Salud Carlos III (ISCIII) and the European Regional Development Fund (FEDER) grant Juan Rodes (JR 16/00020); Ministerio de Sanidad, Política Social e Igualdad, Plan Nacional Sobre Drogas (PNSD) (2012I054); Suport Grups de Recerca AGAUR-Gencat (2017 SGR 316, 2017 SGR 530); Ministerio de Economía y Competitividad (MTM2015-64465-C2-1-R); ISCIII-Redes de Investigación Cooperativa Orientadas a Resultados en Salud (RICORS); Red de Investigación en Atención Primaria de Adicciones (RIAPAd), grant number RD21/0009/0001. The funding agencies had no role in study design, data collection, interpretation, and no influence on the writing.

## Conflict of Interest

FF has received travel grants during the last 3 years from Lundbeck, Otsuka, Indivior, Pfizer, Gilead and Servier; she has also received grant/research support from Indivior and Servier. MT has been consultant/advisor and/or speaker for Gilead Sciences, Merck Sharp & Dohme Corp, Indivior, Mundipharma Pharmaceutics, Servier and Adamed. The remaining authors declare that the research was conducted in the absence of any commercial or financial relationships that could be construed as a potential conflict of interest.

## Publisher's Note

All claims expressed in this article are solely those of the authors and do not necessarily represent those of their affiliated organizations, or those of the publisher, the editors and the reviewers. Any product that may be evaluated in this article, or claim that may be made by its manufacturer, is not guaranteed or endorsed by the publisher.
