# Supplementary material for: BDNF and Cortisol in the Diagnosis of Cocaine-Induced Depression
Source: Front Psychiatry. 2022 Mar 15;13:836771. doi: 10.3389/fpsyt.2022.836771 (PMC8964529; doi:10.3389/fpsyt.2022.836771)
Supplement: Supplementary file 1 [file Data_Sheet_1.docx]

**Table S1.** Psychopharmacological treatment in the study groups by type of antidepressants.

|  | **HC (N=21)** | **MDD**  **(N=6)** | **CUD-induced-MDD (N=9)** | **CUD-primary-MDD (N=16)** | **CUD (N=15)** |
| --- | --- | --- | --- | --- | --- |
| **Tricyclic** | - | 1(16.7) | - | - | - |
| **Heterocyclic** | - | 1(16.7) | 1(11.1) | 2(12.5) | - |
| **NaSSAs** | - | - | 2(22.2) | 1(6.3) | - |
| **NDRIs** | - | 1(16.7) | - | - | - |
| **SSRIs** | - | 2(33.3) | 1(11.1) | 8(50.0) | 1(6.7) |
| **SNRIs** | - | 1(16.7) | 1(11.1) | 2(12.5) | 1(6.7) |
| **Antipsychotics** | - | - | - | - | 1(6.7) |

HC, healthy controls; MDD, major depression disorder; CUD, cocaine use disorder; NaSSAs, Noradrenergic and specific serotonergic antidepressants; NDRI, norepinephrine and dopamine reuptake inhibitors; SSRI: serotonin selective reuptake inhibitors; SNRIs, Serotonin and norepinephrine reuptake inhibitors

**Table S2.** Biological measurements during the Trier Social Stress Test (TSST)

|  | **Heart rate Pre-TSST** | **Heart Rate Post-TSST** | **Heart Rate Post-30-TSST** | **Heart Rate Post-60-TSST** | **Heart Rate Post-90-TSST** |
| --- | --- | --- | --- | --- | --- |
| **HC** | 68.43 ± 11.15 | 76.90 ± 14.56* | 69.86 ± 11.57* | 75.81 ± 10.13* | 77.00 ± 10.58* |
| **MDD** | 73.17 ± 9.30 | 80.67 ± 11.93 | 70.00 ± 9.44* | 77.00 ± 10.86* | 78.17 ± 7.19 |
| **CUD-induced-MDD** | 68.67 ± 10.97 | 75.56 ± 13.40 | 66.44 ± 14.35 | 71.33 ± 13.89* | 73.33 ± 13.82 |
| **CUD-primary-MDD** | 69.94 ± 8.37 | 69.38 ± 7.31 | 67.81 ± 7.67* | 73.38 ± 8.88* | 73.94 ± 7.39 |
| **CUD** | 72.40 ± 12.79 | 73.13 ± 12.69 | 68.29 ± 12.50* | 74.64 ± 13.15* | 78.53 ± 14.65* |
| **One-way ANOVA** | 0.766 | 0.271 | 0.940 | 0.830 | 0.708 |
| ***Paired T-Test <0.05** |  |  |  |  |  |
|  |  |  |  |  |  |
|  |  |  |  |  |  |
|  | **Breath Rate Pre-TSST** | **Breath Rate Post-TSST** | **Breath Rate Post-30-TSST** | **Breath Rate Post-60-TSST** | **Breath Rate Post-90-TSST** |
| **HC** | 17.14 ± 4.15 | 19.43 ± 5.64 | 16.14 ± 4.13* | 17.19 ± 3.66 | 17.19 ± 3.96 |
| **MDD** | 17.67 ± 3.20 | 16.50 ± 2.51 | 16.67 ± 3.72 | 16.67 ± 3.01 | 17.67 ± 2.34 |
| **CUD-induced-MDD** | 17.67 ± 2.83 | 18.33 ± 4.36 | 17.22 ± 2.44 | 17.78 ± 3.53* | 17.33 ± 3.50 |
| **CUD-primary-MDD** | 17.38 ± 3.44 | 16.94 ± 3.47 | 16.50 ± 3.41 | 17.63 ± 3.56 | 16.69 ± 3.11 |
| **CUD** | 18.20 ± 2.01 | 18.67 ± 2.89 | 17.93 ± 2.05 | 18.00 ± 2.14* | 18.07 ± 2.31 |
| **One-way ANOVA** | 0.918 | 0.375 | 0.591 | 0.907 | 0.827 |
| ***Paired T-Test <0.05** |  |  |  |  |  |
|  |  |  |  |  |  |
|  |  |  |  |  |  |
|  | **Systolic Blood Pressure Pre-TSST** | **Systolic Blood Pressure Post-TSST** | **Systolic Blood Pressure Post-30-TSST** | **Systolic Blood Pressure Post-60-TSST** | **Systolic Blood Pressure Post-90-TSST** |
| **HC** | 112.29 ± 12.17 | 126.29 ± 14.07* | 114.67 ± 11.83* | 117.14 ± 11.74 | 116.57 ± 11.79 |
| **MDD** | 128.00 ± 12.95 | 136.50 ± 21.82 | 124.00 ± 10.75 | 128.33 ± 11.36 | 126.50 ± 9.89 |
| **CUD-induced-MDD** | 114.78 ± 13.16 | 123.78 ± 13.83* | 118.56 ± 10.35 | 120.89 ± 10.31 | 118.11 ± 15.15 |
| **CUD-primary-MDD** | 117.63 ± 14.28 | 125.88 ± 16.09* | 118.56 ± 15.37* | 122.50 ± 14.67 | 120.50 ± 13.89 |
| **CUD** | 114.20 ± 14.26 | 119.33 ± 15.53* | 114.00 ± 11.78 | 116.20 ± 8.96 | 117.93 ± 12.02 |
| **One-way ANOVA** | 0.150 | 0.256 | 0.436 | 0.177 | 0.526 |
| ***Paired T-Test <0.05** |  |  |  |  |  |
|  |  |  |  |  |  |
|  |  |  |  |  |  |
|  | **Diastolic Blood Pressure Pre-TSST** | **Diastolic Blood Pressure Post-TSST** | **Diastolic Blood Pressure Post-30-TSST** | **Diastolic Blood Pressure Post-60-TSST** | **Diastolic Blood Pressure Post-90-TSST** |
| **HC** | 66.90 ± 7.14 | 75.43 ± 8.44* | 68.14 ± 7.16* | 68.86 ± 9.63 | 64.67 ± 9.04* |
| **MDD** | 68.50 ± 8.46 | 72.83 ± 15.34 | 67.67 ± 10.84 | 70.50 ± 11.71 | 65.83 ± 8.21 |
| **CUD-induced-MDD** | 70.67 ± 5.29 | 76.11 ± 6.72 | 69.89 ± 4.81* | 73.22 ± 8.63 | 66.22 ± 7.48* |
| **CUD-primary-MDD** | 70.07 ± 10.98 | 76.88 ± 12.81* | 73.19 ± 13.28 | 74.25 ± 11.85 | 71.75 ± 11.46 |
| **CUD** | 68.53 ± 8.81 | 73.93 ± 9.36* | 67.53 ± 7.46* | 70.80 ± 7.97 | 67.57 ± 6.06* |
| **One-way ANOVA** | 0.767 | 0.904 | 0.417 | 0.548 | 0.206 |
| ***Paired T-Test <0.05** |  |  |  |  |  |

HC, healthy controls; MDD, major depression disorder; CUD, cocaine use disorder.
